# Assessment of a venous thromboembolism prophylaxis shared decision-making intervention (DASH-TOP) using the decisional conflict scale: a mixed-method study

**DOI:** 10.1186/s12911-023-02349-3

**Published:** 2023-11-06

**Authors:** Montserrat León‑García, Brittany Humphries, Pablo Roca Morales, Derek Gravholt, Mark H. Eckman, Shannon M. Bates, Nataly R. Espinoza Suárez, Feng Xie, Lilisbeth Perestelo‑Pérez, Pablo Alonso‑Coello

**Affiliations:** 1 Iberoamerican Cochrane Center, Biomedical Research Institute Sant Pau (IIB Sant Pau), Barcelona, Spain; 2 Department of Pediatrics, Obstetrics, Gynaecology and Preventive Medicine, Universidad Autónoma de Barcelona, Barcelona, Spain; 3 Knowledge and Evaluation Research Unit, Department of Medicine, Mayo Clinic, Rochester, MN USA; 4 Department of Health Research Methods, Evidence and Impact, McMaster University, Hamilton, ON Canada; 5 Faculty of Health Sciences, Universidad Villanueva, Madrid, Spain; 6 School of Health Sciences, Valencian International University, Valencia, Spain; 7 Division of General Internal Medicine and Center for Clinical Effectiveness, University of Cincinnati College of Medicine, Cincinnati, OH USA; 8 Department of Medicine, McMaster University, Hamilton, ON Canada; 9 VITAM Research Center for Sustainable Health, Quebec City, Canada; 10 Faculty of Medicine, Université Laval, Quebec City, Canada; 11 Centre for Health Economics and Policy Analysis, McMaster University, Hamilton, ON Canada; 12 Evaluation Unit (SESCS), Canary Islands Health Service (SCS), Tenerife, Spain; 13 Network for Research On Chronicity, Primary Care, and Health Promotion (RICAPPS), Tenerife, Spain; 14 CIBER of Epidemiology and Public Health, CIBERESP, Madrid, Spain

**Keywords:** Decision aids, Informed decision choices, Decisional conflict, Shared decision-making, Values and preferences and decision analysis

## Abstract

**Background:**

Venous thromboembolism (VTE) in pregnancy is a major cause of maternal morbidity and death. The use of low-molecular-weight heparin (LMWH), despite being the standard of care to prevent VTE, comes with some challenges. Shared decision-making (SDM) interventions are recommended to support patients and clinicians in making preference-sensitive decisions. The quality of the SDM process has been widely assessed with the decisional conflict scale (DCS). Our aim is to report participants’ perspectives of each of the components of an SDM intervention (DASH-TOP) in relation to the different subscales of the DCS.

**Methods:**

Design: A convergent, parallel, mixed-methods design.

Participants: The sample consisted of 22 health care professionals, students of an Applied Clinical Research in Health Sciences (ICACS) master program.

Intervention: We randomly divided the participants in three groups: Group 1 received one component (evidence -based information), Group 2 received two components (first component and value elicitation exercises), and Group 3 received all three components (the first two and a decision analysis recommendation) of the SDM intervention.

Analysis: For the quantitative strand, we used a non-parametric test to analyze the differences in the DCS subscales between the three groups. For the qualitative strand, we conducted a content analysis using the decisional conflict domains to deductively categorize the responses.

**Results:**

Groups that received more intervention components experienced less conflict and better decision-making quality, although the differences between groups were not statistically significant. The decision analysis recommendation improved the efficacy with the decision-making process, however there are some challenges when implementing it in clinical practice. The uncertainty subscale showed a high decisional conflict for all three groups; contributing factors included low certainty of the evidence-based information provided and a perceived small effect of the drug to reduce the risk of a VTE event.

**Conclusions:**

The DASH-TOP intervention reduced decisional conflict in the decision -making process, with decision analysis being the most effective component to improve the quality of the decision. There is a need for more implementation research to improve the delivery of SDM interventions in the clinical encounter.

**Supplementary Information:**

The online version contains supplementary material available at 10.1186/s12911-023-02349-3.

## Background

Venous thromboembolism (VTE) in pregnancy causes approximately 1.5 to 2% of maternal deaths during pregnancy and the postpartum period, and is a major cause of maternal morbidity [1, 2]. The use of thromboprophylaxis with low-molecular-weight heparin (LMWH) is the standard of care in women who have a history of VTE that was unprovoked or was associated with a hormonal risk factor or a prior VTE associated with a nonhormonal temporary provoking risk factor and no other risk factors [3, 4]. Prophylaxis reduces the risk of recurrent VTE by almost 75% [5]; it is safe for the fetus, compared to other alternatives, such as aspirin or unfractionated heparin. However, it is difficult to administer, may cause pain, is expensive, and the certainty of the available evidence of its efficacy is low [6–8]. Therefore, this treatment does not constitute the ‘single best option’ in at risk pregnant women, and clinical guidelines encourage clinicians to consider women’s preferences when assessing the trade-offs between alternatives (prophylaxis with daily injections of LMWH vs. no prophylaxis) [4, 5, 8].

Shared decision-making (SDM) is the gold-standard approach when dealing with preference sensitive decisions [9, 10]. The International Patient Decision Aids Standards (IPDAS) collaborative suggests the use of different techniques (e.g., provision of evidence-based information, improving healthcare professional communication, and value elicitation exercises to clarify patient preferences) to improve patient engagement in decision-making and to support the process of SDM in achieving a high-quality informed decision [10, 11].

The international collaborative group DASH-TOP (Decision Analysis Shared decision-making Thromboprophylasis Of Pregancy) [12], is developing a SDM intervention to support women and their clinicians in making decisions about the best strategy to prevent recurrent VTE during pregnancy. This SDM intervention contains three components: 1) evidence-based information about the risks and benefits of taking LMWH to prevent VTE; 2) values elicitation exercises to explore women’s preferences for each of the four health states relevant to this decision (i) inconvenience of using LMWH during pregnancy; (ii) major obstetrical bleed; (iii) Deep Venous Thrombosis (DVT) in pregnancy; and (iv) Pulmonary Embolism (PE) in pregnancy; and 3) a decision analysis (DA) model that presents which alternative has better *expected value* (i.e., quality-adjusted life year (QALY)) considering individualized data on the patient’s risks and preferences toward different health states [13]. These components have been used successfully to support SDM resulting in improvements in the quality of the decision-making process [10, 14, 15]. However, despite decision analysis being a technique used in SDM, the process of decision-making while using this method is not yet fully understood, leading to a lag in implementation of this technique [13]. The DASH-TOP group tested the intervention, in a pilot sequential mixed-method study [16], with women who were planning pregnancy or pregnant at the time of the study, and were at risk of presenting a recurrent VTE event during their pregnancy. In parallel, with the aim of assessing how this SDM intervention can be implemented, we conducted the present study with health care professionals of different backgrounds that were enrolled in a master program to retrieve their thoughts on the feasibility of implementing a SDM intervention containing these three components. While different instruments have been used to evaluate SDM interventions [17], there are still challenges in measuring the quality of the decision-making process [18]. The Decisional Conflict Scale (DCS) is the instrument most commonly used for this purpose [10, 15]. The DCS contains different subscales [19, 20] that assess different domains of the SDM process (informed, values clarity, support, uncertainty and effective decision-making) [19]. The DCS is mostly used to assess the effectiveness of decision aids vs. usual care [10, 21]. However, there is a scarcity of literature using the DCS to compare several SDM interventions [22], and using its subdomains to evaluate the process through which a decision-making support technique function [23].

Our research question was: What are participants’ perspectives of each of the components of the DASH-TOP intervention in relation to the different subscales of the DCS?

## Methods

### Research design

We adopted a convergent, parallel, mixed-methods design [24, 25], including quantitative methods to elicit the quality of the decision-making process using the DCS, and an open-ended questionnaire to qualitatively explore the deliberation process that occurred during each component of the SDM intervention. We then integrated the findings from the two sets, and investigated the relationship between the qualitative and quantitative sets. We used a joint display to present the integration of the results [25, 26]. We followed the standard guidelines for Good Reporting of A Mixed Methods Study (GRAMMS) [27].

### Participants

We recruited postgraduate health care professionals who were enrolled in the Applied Clinical Research in Health Sciences (ICACS) master program at Universidad Autónoma de Barcelona during the 2020–2021 and 2021–2022 academic years. Participants were simultaneously working as health care professionals. Subjects were informed about the study and gave written consent to participate. The study was approved by the ethical committee board of Hospital de la Santa Creu i Sant Pau (IIBSP-TDC-2018-02) in accordance with the Declaration of Helsinki.

Data were collected using convenience sampling. We recruited participants, collected the data and analyzed it for each academic year, continuing recruitment the following year until saturation (no new themes emerging from the analysis) was reached [28, 29]. After discussion among two members of the research team (ML-G and PA-C), saturation was determined to be obtained after the second year and, therefore, recruitment concluded.

### Intervention procedure

**Fig. 1:**
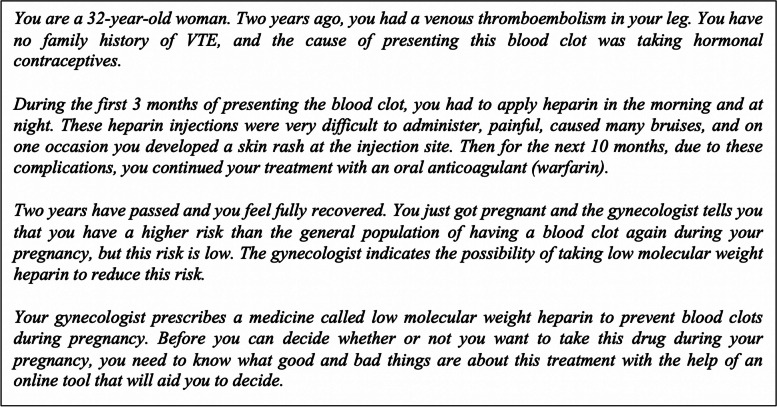
Study case

The intervention was delivered as part of the master program’s module on “Values and Preferences” taught by two of the authors (ML-G, PA-C). It was conducted using a real-time video conferencing platform (Zoom) due to the COVID-19 pandemic. All participants were presented with a case study (Fig. 1).

**Fig. 2:**
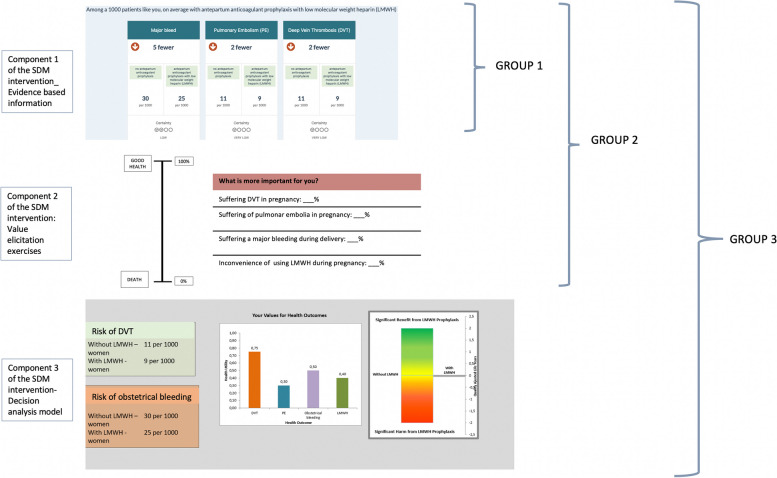
Allocation procedure

Participants were randomly divided (sorted alphabetically based on their last name) into three different virtual groups. We followed an incremental design with varying exposure to the different components: group 1 was exposed to the first component of the SDM intervention, group 2 to two components, and group 3 to all three components. Figure 2 represents the intervention components and group allocation.

**Fig. 3:**
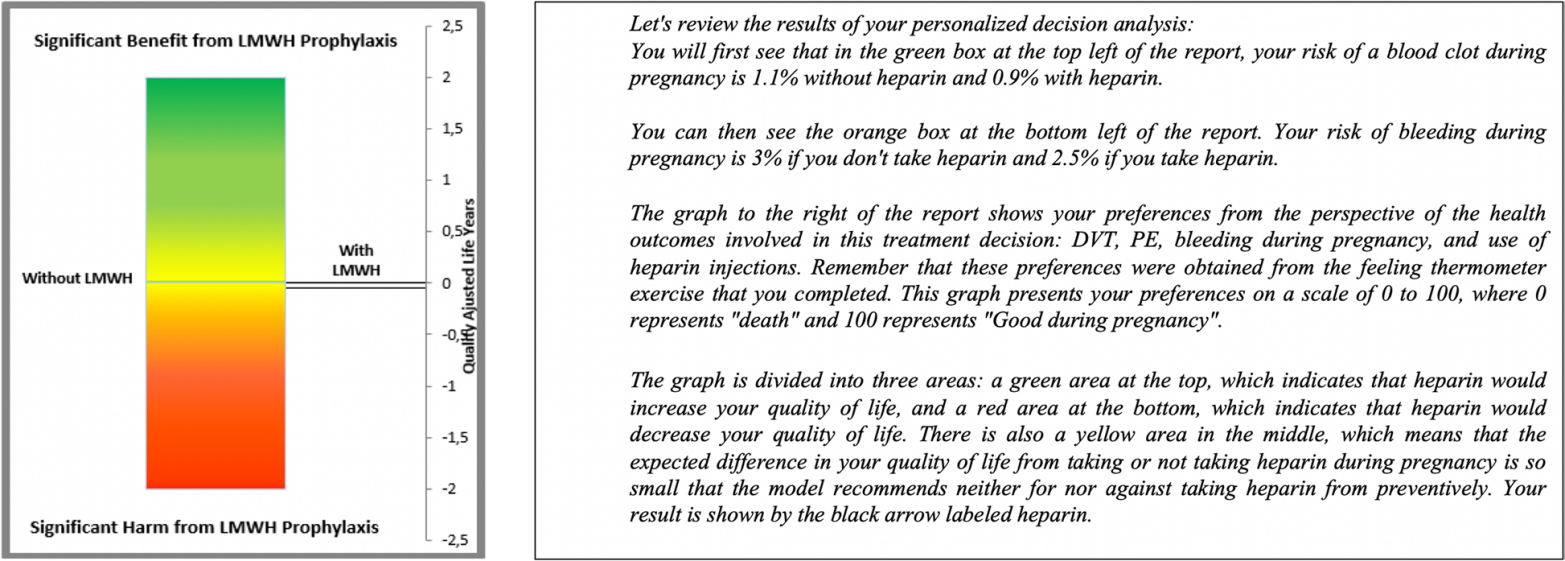
Decision analysis recommendation

The virtual room was divided into three rooms to ensure blinding of participants and to avoid interference between groups. Researchers delivered each component independently to each group as follows:

I) Group 1: This group received only the evidence-based information component of the intervention. Participants were presented with scenarios for four clinical situations (health states): suffering DVT in pregnancy, suffering PE in pregnancy, suffering major obstetric bleeding (MOB), and the inconvenience of using LMWH in pregnancy; all of which had been previously validated [6, 7]. This information was provided with graphical representations.
II) Group 2: This group first received the evidence-based information, followed by two value elicitation exercises: 1) a ranking exercise in which participants were asked to rank the four health states, from most to least preferred; and 2) a visual analogue scale (VAS) in which participants placed each health state along a “feeling thermometer” that represents the importance of suffering each health state for them, on a scale of 0 (death) to 100 (well in pregnancy). The percentage value placed on the feeling thermometer matches the value filled on the table.
III) Group 3: This group received all three components of the intervention: evidence-based information, value elicitation exercises and a decision analysis recommendation. Their value ratings obtained from the VAS exercise were inputted into a decision analytic model. The decision analytic model is a Markov state transition model developed for the DASH-TOP intervention [7, 12, 30] that examines the two treatment options under consideration: using LMWH prophylaxis versus no LMWH as prophylaxis for prevention of recurrent VTE during pregnancy. This mathematical model uses women’s age and risk of VTE, combined with their value ratings and probabilities of suffering each health state during pregnancy, to estimate the QALYs for each treatment option [31]. The treatment with the greatest expected QALYs is presented as the decision analysis recommendation. Participants received the results and recommendation of their personalized decision analysis using a graphical representation, accompanied by a written explanation as shown in Fig. 3.

Participants were asked how long it took them to complete the intervention. After completing the intervention, participants were asked to complete the DCS and then respond to an open-ended questionnaire regarding their perceptions of the different components and the decision-making process from their perspective as health professionals visiting a woman with this clinical situation (Fig. 1).

### Data collection

**Fig. 4:**
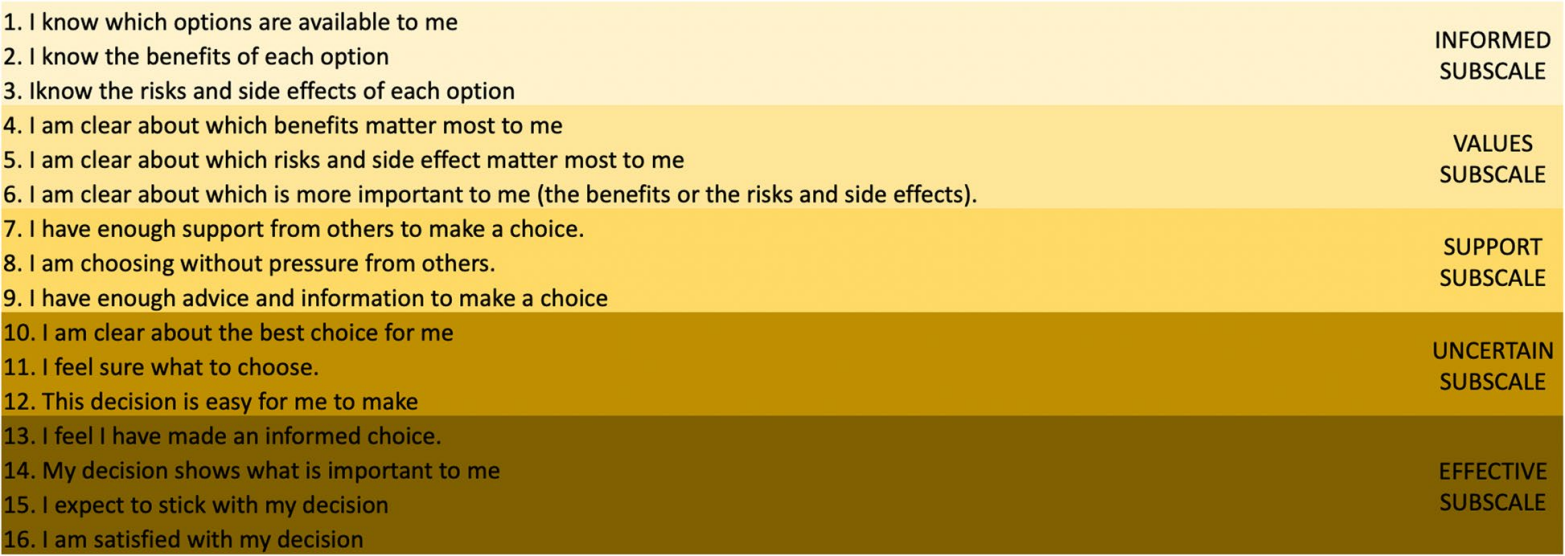
Decisional conflict subscales

We used the DCS instrument [19, 20] to evaluate the quality of the SDM process. This scale contains 16 items divided in five subscales: i) informed; ii) values clarity; iii) procedure support; iv) uncertainty; and v) effective decision. Scores for each item range from 1 (strongly agree) to 5 (strongly disagree), with high scores indicating higher decisional conflict. Research in general health care has established cut-off points for scores that show no decisional conflict (DC) (< 25), a low level of DC (> 25-<37.5) and a high level of DC (> 37.5) [19, 21, 32, 33]. Generally, DCS scores < 25 are associated with decisions with minimal conflict and scores > 37.5 demonstrate high conflict and are associated with uncertainty about the best action or decision delay [15]. Figure 4 represents the specific items from the DCS, corresponding to each subscale.

For the qualitative data, all participants were asked to respond to six open-ended questions to assess the factors involved in the decision-making process, whether the information was appropriate, and how they balanced harms and benefits of the treatment alternatives. Additionally, groups 2 and 3 were asked to describe their experience and perceptions of the value elicitation exercises, and group 3 was also asked to describe the usefulness of the decision analysis to make a decision and their confidence with the decision-making process. In Supplementary material 1, we provide the full script of the open-ended questionnaire, noting the group to which questions correspond.

### Data analysis

#### Quantitative analysis

We conducted descriptive analyses to report the mean and standard deviation (*SD*) for the total DCS and each subscale. No missing values were found. Given the small sample size, and after testing the basic assumptions (i.e., normality, homogeneity of variances, and independence), Kruskal–Wallis *H* non-parametric test was used to analyze the differences in DCS between the three groups. SPSS 26 for Windows was used for all quantitative analyses.

#### Qualitative analysis

For qualitative data, we recorded data from the individual questionnaires and introduced it in a Microsoft Excel file. Two members of the research team (ML-G and B-H) independently coded the transcripts by conducting a content analysis using the decisional conflict subscales to deductively categorize the responses. For example, we collected responses related to how clear it was to decide the benefits and drawbacks of taking medication, and matched it with the values clarity subscale.

#### Data integration- mixed method analysis

Quantitative and qualitative results were merged; this process can be found in Supplementary material 2. We presented the data by displaying the mixed-methods graphically, showing quantitative scores and qualitative quotes side-by-side, to provide a comprehensive view of the perceptions of the decision-making process. We then presented in a tabular format how factors of the SDM intervention contributed to an increase or decrease in decisional conflict for each DCS subscale. Finally, we reported the strengths and challenges of implementing each of the intervention components to support the SDM-process. Four members of the team (M-LG, B-H, L-PP, P-AC) with experience using SDM interventions discussed and agreed on the interpretation of these findings.

## Results

We approached 44 health master students, 22 (50%) of whom agreed to participate (16 from the first academic year and six from the second). Reasons for not participating were not collected. The majority of participants were women (72,7%). All students were working as health professionals of the following disciplines: eight (36%) nurses; five (23%) physicians (surgeon, pediatrician and general practitioners); four (18%) physical or occupational therapists; two psychologists (9%); two dentists (9%); and one (5%) microbiologist. Supplementary material 2 describes participants’ characteristics. Participants were randomly assigned into the three groups: G1 Evidence-based information component (*n* = 7); G2 Value elicitation exercises component (*n* = 7); and G3 Decision analysis (*n* = 8). Participants in G1 spent on average 18 min to complete the intervention; for G2, it was 26 min; and for G3, it was 33 min.

### Decisional conflict scale

Data collection and analysis is accessible in Supplementary material 2.

#### Total DCS

Overall, decisional conflict scores were low (mean = 24.22; SD = 14.47) for all three groups. For the total DCS, Group 1, who received only the evidence-based information intervention, showed higher conflict score (mean = 25.89; SD = 17.35; low conflict), and two (28.6%) of the participants were unsure of what their final decision would be after completing the intervention. Those that also completed the value elicitation exercises (Group 2) presented a lower conflict (mean = 24.55; SD = 17.01; low conflict). Only one participant (14.3%) felt unsure of what their final decision would be after completing the intervention. Furthermore, those that also received the decisional analysis model result (Group 3) had the lowest level of conflict (mean = 22.46; SD = 10.76); no conflict), and none of the participants felt unsure of what their final decision would be after completing the intervention. However, despite these trends, no significant differences between the intervention groups were observed (*H*_(2)_ = .06; *p* = .97).

#### DCS subscales

Table 1 presents participants’ perspectives of each of the components of the SDM intervention in relation to the different subscales of the DCS. We present a joint display of the scores for each subscale per group (represented with box and whisker plots) and, in parallel, the qualitative findings with a representative quote. Those instances in which there was discrepancy between the DCS score and what was reported in the open-ended questionnaire are in bold font. As follows, we describe the interpretation for each subscale.

**Table 1:**
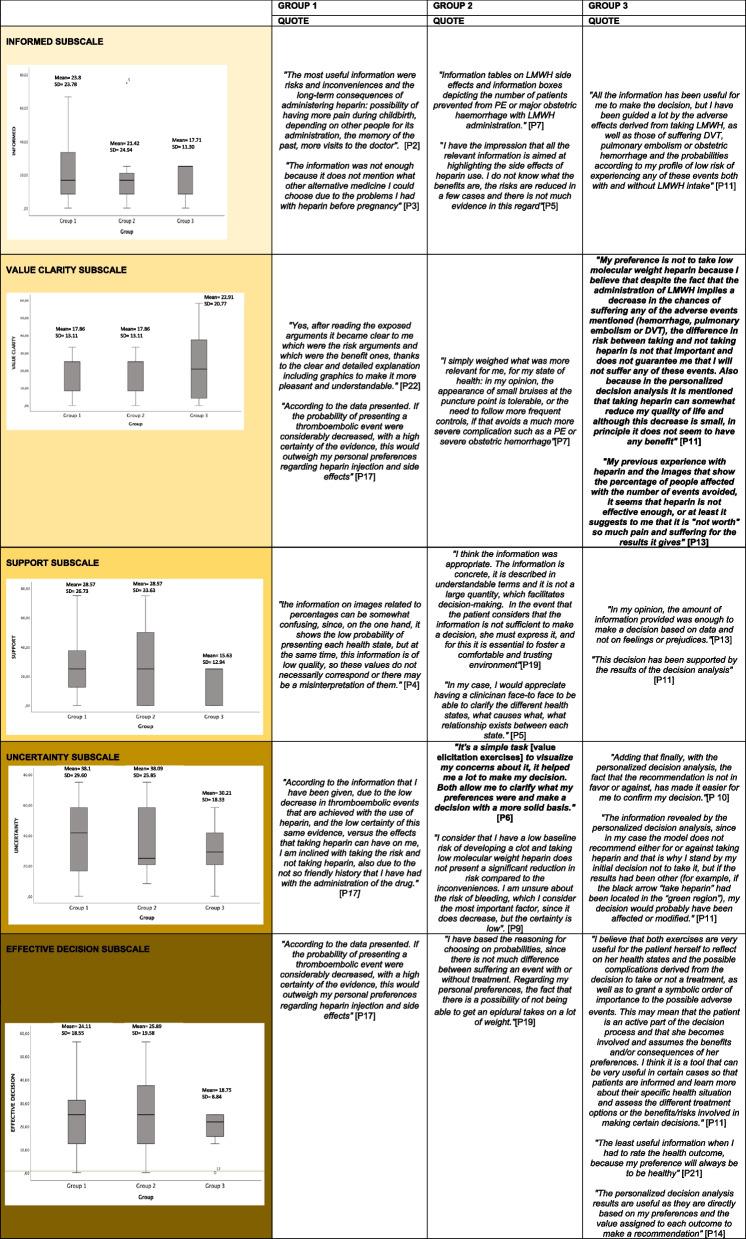
Joint display of quantitative and qualitative data for each DC subscales

##### Informed subscale

This subscale informs about the presence of conflict regarding the knowledge participants have around the benefits and risks of the options. The mean score for this subscale showed no conflict (mean = 20.83; SD = 19.71) and there were no significant differences between groups (*H*_(2)_ = .22; *p* = .89). The results follow the same pattern as the total decisional conflict score showing higher conflict for Group 1 than Group 2, with Group 3 showing the least conflict. For all groups, there was concordance between the quantitative and qualitative results.

Group 1 felt that their decision was mainly informed by the content of the health states (43%), the graphical representation of the risks and benefits of taking LMWH (43%), and their previous experience (29%). Some participants (29%) showed some conflict (> 25-<37.5) because they didn’t know if other options were available (like oral anticoagulants). In Group 2, all participants (100%) were aware of the options, and exclusively referred to the graphical representations to inform about the benefits and risks of each option. There was one outlier that showed a higher conflict (> 37.5) in comparison with the rest of the group; this participant reported not having enough information about the benefits of taking LMWH. Group 3 felt they knew the risks and benefits of each option due to: the information provided by the health states (50%); the graphical representations (38%), and previous experience (25%).

##### Value clarity subscale

The value clarity subscale informs on how clear it is to decide which benefits and risks matter more and how clearly it is to know what is most important (the benefits or the risks). This subscale had the lowest conflict score among all of the DCS subscales (mean = 19.70; SD = 15.76) and no significant differences were found between groups (*H*_(2)_ = .24; *p* = .89). Group 1 and Group 2 showed the same level of conflict, and the quantitative and qualitative results were in concordance. Group 3 showed more conflict than the other two groups, and there was a discordance between the quantitative and qualitative sets.

All participants in Group 1 agreed that the information provided graphically about the probability of a VTE event or bleeding event occurring was clear enough to assess what matters most to them (benefits or risks). One participant (14%) added that the low certainty of the evidence was confusing when carrying out the tradeoffs of the decision. In Group 2, the majority (57%) used the content of the health states to know what benefits and what risks matter most; some participants (43%) highlighted the use of the value elicitation exercises to help them weigh the pros and cons of each health state and clarify what was more important. In Group 3, there was a discrepancy between the quantitative and the qualitative data; while this group showed the highest conflict for this subscale (mean = 22.91; SD = 20.77) of all three groups, their qualitative perceptions were that it was clear to them what matters most, risks or benefits. In addition, half of the participants (50%) reported the decision analysis model was useful and helpful to resolve imbalances and make a final decision.

##### Support in the decision-making subscale

This subscale provides information about the support in the process of decision-making and contains three items. The first two items of the subscale were excluded from the analysis as they were questions around “*the support given by others*” and “*if there was any pressure by others when making the decision*”. We only included the item regarding the amount of advice and information about the choices. The mean score for this subscale showed no conflict (mean = 23.86; SD = 24.97) and there were no significant differences between groups (*H*_(2)_ = 1.7; *p* = .43). Group 1 and Group 2 showed low conflict (25-37,5) and Group 3 showed no conflict score (< 25). For all groups, there was concordance between the quantitative and qualitative results.

The majority of participants in Group 1 (86%) agreed that the amount of information was adequate; however, the low certainty of the evidence suggests the need for further research on the efficacy of the drug. Most of the participants in Group 2 (71%) stated that the information was adequate. Two participants in Group 2 (29%) that had higher levels of conflict (> 37,5) expressed the need to receive support from their health professional to understand the information given on health states and address other concerns the women could present in this context. Group 3 showed no conflict in this subscale and the majority agreed (75%) that the amount of the information. Two participants reported that the information given by the decision analysis supported their decision.

##### Uncertainty subscale

This subscale reflects on certainty of which choice is best for the patient and how easy it is to make the decision. This subscale showed high conflict score (mean = 35.23; SD = 23.84), with Group 1 and Group 2 presenting higher conflict than Group 3; however, there were no significant differences between groups (*H*_(2)_ = .27; *p* = .87). Quantitative and qualitative results were concordant for Group 1 and 3, but discordant for Group 2.

In Group 1, the high level of conflict was attributed to high uncertainty regarding a perceived low efficacy of the drug and the low certainty of the evidence (71%). Also, some participants (57%) found it difficult to decide due to the adverse effects presented in their previous experience. Group 2 participants, except one (14%), expressed certainty about their choice, and they all reported that the value elicitation exercises eased the weighing of the benefits and drawbacks of each option. In addition, two participants (29%) noted the low quality of the evidence that was used to inform the outcomes as a basis for their uncertainty. Group 3 participants highlighted that the provided evidence-based information reduced their uncertainties with the decision, and it was clear what choice was best: for the majority (88%), this decision was not to take LMWH due to the low benefits in reducing the risk of a VTE event. Two participants reflected on the decision analysis component and gave discordant perspectives: while one thought that not having a clear recommendation on whether to take LMWH (recommendation shown in the yellow area) introduced uncertainty in the decision-making process (P11 with a high conflict > 37,5), the other thought that this made it easier to confirm their decision, and that there was not a “correct option” (P10 with low conflict > 25-<37.5).

##### Effective decision subscale

This subscale reflects the final decision and whether choices were well-informed, which points to the level of satisfaction with the process. The mean score for this subscale showed no conflict (mean = 22.73; SD = 15.61), and there were no significant differences between groups (*H*_(2)_ = .69; *p* = .71), although Group 1 and Group 2 presented higher conflict than Group 3. For all groups, there was concordance between the quantitative and qualitative results.

Group 1 reported conflict on how effective the decision-process was mainly because the data was not adequately informed. For example, one participant explained that the low efficacy of the drug and low quality of the evidence hinder the process of decision-making and deciding what is most important. Group 2 showed a slightly higher level of conflict compared to Group 1 because some participants (29%) felt that the value elicitation exercises did not include aspects that were important in their decision-making (such as long-term effects). In Group 3, we found there was better satisfaction (no conflict < 25) with the process compared to the other two groups (> 25). Some participants (50%) highlighted the role of the value elicitation exercises to feel more involved in the decision-making process; however, other participants (25%) thought the exercises could be confusing (low conflict > 25-<37.5)), especially the VAS (for them, it is always important to be in ‘good health’). Although all participants from Group 3 responded that the decision analysis was useful to confirm their decision, half of the participants (50%) would not trust the decision analysis model alone to make the final decision; they would prioritize their own desires and would appreciate the support of their health professional.

In Table 2 we highlighted the key takeaways of these results, by presenting which factors of the SDM intervention identified by participants may contribute to decrease and increase the decisional conflict for each DCS subscale.

**Table 2:** Summary of findings

DCS SUBSCALE (meaning)	Factors of the SDM intervention contributing to decrease decisional conflict	Factors of the SDM intervention contributing to increase decisional conflict
INFORMED (knowledge of the decision)	♣ Knowledge on the condition ♣ Previous experience with the treatment ♣ Graphical representation to deliver information on risk reduction	♣ Not enough information on benefits and harms
VALUE CLARITY (how clear preferences are in the decision)	♣ Value elicitation exercises ♣ Decision analysis	♣ Not having a health professional to clarify recommendation of the decision analysis
SUPPORT IN THE DECISION-MAKING (if the amount of information provided is enough to support their decision-making process)	♣ Evidence-based information ♣ Decision analysis	♣ Low efficacy of the treatment for risk reduction ♣ Need of health professional to support the decision-making process
UNCERTAINTY (how sure participants are with the decision and how easy it was to make the decision)	♣ Value elicitation exercises	♣ Low certainty of the evidence ♣ Decision analysis recommendation not on favor or against the treatment
EFFECTIVE DECISION (adequately and appropriately informed decision-making, and satisfaction with the decision-making process)	♣ Value elicitation exercises ♣ Decision analysis	♣ Low quality of the evidence

In Table 3 we report the strengths and challenges of implementing each of the SDM intervention components reported by participants from their perspective as a health professional.

**Table 3:** Strengths and challenges of each SDM intervention component

Intervention component	Strengths	Challenges
**Evidence-based information**	**-** The description of health states is very relevant to identify what factors inform the decision-making process. **-** Graphical representations and figures of risks and benefits are important to make them aware about the choices, and clarify what the options are.	**-** The factors that were contributing to the decision were arbitrary, dependent of participants preference (e.g. some people were more concerned about the use of epidural at birth, and others about the need to do something to avoid a VTE event), and influenced by their previous experiences with the condition or drug. **-** The low certainty of the evidence hinders the process of decision-making. **-** The low efficacy of a drug (small risk reductions) makes it difficult to decide what is most important. **-** The level of health literacy of participants influences the understanding and usefulness of the decision analysis recommendation.
**Value elicitation**	-Exercises are useful to assess whether risks are more important than benefits, through a weighing process. - Exercises foster participation of participants in the decision-making process.	- Having specific health states to rate what is more important might restrict the decision-making process or aren’t comprehensive enough, and leave aside other factors that may contribute to the decision (e.g. long-term consequences).
**Decision analysis**	- Reduces overall decisional conflict - It is useful to reinforce and/or confirm their decision making the process of decision-making more precise. - It improves satisfaction as their individual preferences are considered and linked to a recommendation on what is the best option.	**-** Having a section where the options neither favor nor are against the intervention can be confusing (they fall in the yellow area). **-** Would not rely on the decision analysis recommendation uniquely, and need the support of the health professional in the decision-making process - Need to provide information in a timely manner.

## Discussion

### Main findings

In this study, we aimed to report participant’s perspectives on using an SDM intervention to reduce decisional conflict of a preference-sensitive decision; the use of thromboprophylaxis during pregnancy. Our main finding is that all three components of the SDM intervention (evidence-based information, value elicitation exercises and decision analysis) reduce the overall decisional conflict. We found no decisional conflict (DCS < 25) for the overall DCS score and the majority of the subscales, for all three groups; however, this trend was not statistically significant. We found a high level of decisional conflict (> 37.5) for the uncertainty subscale due to the low certainty of the evidence, however value elicitation exercises showed to have the potential to reduce the uncertainty of the process.

#### Effect of the SDM intervention components on DCS

Evidence-based information reduced conflict in the decision-making process. However, when the information is based on low quality evidence, uncertainty increases. A similar study conducted in Spain [34], assessing a decision aid for breast cancer screening also noted the importance of providing evidence-based information to improve decision-making: women positively value receiving information regarding the benefits and harms of breast cancer screening. As in previous studies [35, 36], we found that graphical representation of risks and benefits using pictograms showing the number of people experiencing an event with and without medication, reduces decisional conflict by clarifying the numerical information provided. Participants in our study also noted that patients’ health literacy should be assessed to ensure adequate understanding of the information. As shown by several authors who explored the relationship between health literacy and DCS, a better understanding of health information can significantly decrease decisional conflict [37].

Value elicitation exercises were useful to understand what is most important (risks or benefits) in a decision, thus supporting and facilitating the weighing activity (pros and cons) in the decision-making process. These exercises also reduced uncertainty in the decision-making process by helping participants better clarify (‘what choice is best for me’) their decision. This finding is consistent with IPDAS recommendations [11, 15]. Furthermore, exploring patients’ values and preferences contributes to patient engagement in the decision-making process, improving self-efficacy. Supportive of these findings, a recent cross-sectional study [37] assessing factors contributing to a lower decisional conflict found that respondents, who reported higher ability to actively engage and participate in the decision-making process, had lower decisional conflict.

The provision of a decision analysis recommendation decreased uncertainty (lowest level of decisional conflict for the uncertainty subscale) and improved self-efficacy with the decision process. It helped tip the balance of pros and cons, helping participants to be more confident with their decision. In addition, participants noted the need for health professionals when implementing the decision analysis technique in the clinical encounter, to support the cognitively-demanding activity of integrating the evidence with their preferences [38–40]. On this regard, Dumont and colleagues [41] have referred to the use of decision analysis, as a decision support technique that promotes a meaningful dialogue between providers and patients on preferences, options, concerns, risks and benefits, leading to an informed and more satisfactory decision for both parties.

#### Decisional conflict scale as an instrument to assess the SDM process

In the context of SDM, decisional conflict is one of the most frequently reported outcomes in studies assessing decision support interventions [10, 18, 21, 32], and the DCS appears to be an optimal instrument to measure the quality of the process [33]. All the subscale items are in line with other instruments used to measure the quality of SDM interventions, such as the widely used SDM-Q-9, MAPPIN’SDM, and OPTION [17]. However, a review assessing the quality of the SDM process highlighted that their common usage does not imply that these measures have adequate congruence with the conceptualization of SDM used to develop the intervention, as they do not necessarily capture the effect of the interactions among the decision-makers (i.e. patients, clinicians, family) [23]. As seen in our study, the support subscale (how supported do patients feel in the decision-making process) needs further attention, especially the role of health professionals to support the process. For example, the CollaboRATE scale [42] further explores the support from clinicians in decision-making with items like ‘how much the provider listened to them about their health issue’. The need for health professionals as decisional partners was also highlighted by Legaré and colleagues [32] when developing a modified decisional conflict scale (D-DCS) with the aim of evaluating the decision-making process in SDM encounters, concluding that the patient-clinician relationship affects the quality of the decision. Furthermore, there is a need to understand the impact of peer pressure on decision-making. For example, in our decision context, some authors [8, 43] have reported that the opinions and support from the husband of a pregnant woman going through this decision, as well as experiences from other women who went through this same condition may be important to support them.

The different DCS subscales have normally been compared in relation to usual care [9, 10, 21, 44], less frequently when comparing SDM interventions [22, 23], or for decision analysis as an intervention for SDM [13]. In a study [44] evaluating the DCS for measuring the quality of end-of-life decisions, authors found significant differences in DCS scores between usual care (higher DCS scores) and the intervention (containing an evidence-based component and value elicitation exercises), and these were due to factors contributing to uncertainty and the efficacy of their decisions. They highlight some of the factors contributing to high uncertainty; feeling uninformed, feeling unclear about personal values, and feeling unsupported. Our study also showed that the subscale showing high conflict between groups was the uncertainty subscale (how clear and sure do patients feel about what to choose) and was attributed to the low certainty of the evidence and the support from others (specially clinicians) in the decision-making process. Despite this, value elicitation exercises did help clarify personal values. Other authors [13, 38–40] have also reported on the contribution of decision analysis to support SDM and improve the uncertainty and effectiveness of the process; as Robinson and colleagues [39] explain: decision analysis was of value as it seeks to create a rational framework for evaluating complex medical decisions and to provide a systematic way of integrating potential outcomes with probabilistic information. However, our findings, as well as a scoping review on SDM containing decision analysis [13] highlighted the difficulties on how to implement decision analysis recommendations in clinical decision-making. Our results reveal that some of these challenges are related to how to present recommendations in the clinical encounter, and to deliver the information in a timely manner.

### Limitations and strengths

Our sample consisted of students enrolled in a master program and, therefore, we cannot extrapolate our results to the target population of women with a previous VTE event. This limitation was partly due to the COVID-pandemic, which hindered the recruitment of participants [45]. Therefore, we conducted this study in parallel to a study our team was developing with the target population [12, 16]. However, our focus was to understand the quality of the decision-making process (i.e., how decisional conflict increased or decreased) with respect to each SDM intervention component. To this end, because our participants were active health professionals, they had helpful insights to understand the potential sources of conflict that may arise when implementing SDM interventions in a clinical context. The randomization method we used (sort alphabetically by last name) did not ensure having symmetrical groups and it would have been useful to have assessed the baseline knowledge on gynecology and obstetrics of our participants to ensure the comparability of the three groups [46, 47]. However, participants were invited to self-report to what extent their knowledge or experiences influenced their decision-making, and provided reflections about similar examples in their clinical practice where they deal with preference sensitive decisions. In addition, we acknowledge the small sample size of our study as well as the different specialties of the health professionals included in our study and not having target clinicians such as gynecologists, obstetricians or hematologists for the decision assessed. Despite this limitation, we observed trends that were consistent with the qualitative findings.

Using a mixed method approach, and presenting the data in a joint manner, are some of the main strengths of our study. As other authors [25, 26, 48] have also reported, mixed-methods designs facilitate the understanding of complex phenomena and overcome the limitation that quantitative data have in understanding complex decision-making processes.

### Implications for practice and research

We highlight four main implications of our study that should be addressed in future research and clinical practice:

- First, high certainty of the evidence is needed to construct decision aids that aim to improve informed decision-making. This is especially important and challenging when there is equipoise regarding the efficacy of alternative treatments. Hence, more studies with larger sample sizes are needed to assess women’s values and preferences for the use of LMWH in pregnancy, thus providing high quality evidence to develop SDM interventions.
- Second, we highlight the importance of including components that specifically explore patients’ values and preferences, such as value elicitation exercises, to reduce decisional conflict. Simple exercises exploring factors such as their previous experience with the condition or treatment, should be included in the development of SDM interventions.
- Third, decision analysis has the potential to add value by reducing uncertainty and improving the efficacy and satisfaction with the SDM process. The cognitive reasoning activity of balancing pros and cons could be eased by an algorithm (decision analysis) that combines preferences with evidence. More implementation research is needed on how to deliver the decision analysis recommendation in clinical practice.
- Fourth, it is essential to assess the interaction between patient and health professional, as well as include health professionals in the development of SDM tools [49] to better understand the feasibility when implementing them in the clinical encounter [50].

## Conclusion

All three components of the DASH-TOP intervention (evidence-based information, value elicitation exercises and decision analysis) can reduce decisional conflict and improve the quality in the decision-making process. The presentation of patient-tailored decision analytic results helped subjects better understand tradeoffs between risks and benefits of treatment alternatives, and provided an added value to the decision-making process. However, presenting results in real-world clinical settings remains a challenge.

### Supplementary Information

**Additional file 1: Table S1.** Open-ended questionnaire script for qualitative data collection.

**Additional file 2: Table 1.S2.** Participant Characteristics. **Table 2.S2.** Quantitative Data Collection. **Table 3.S2.** Quantitative Data Analysis. **Table 4.S2.** Qualitative Data Collection. **Table 5.S2.** Qualitative Data Analysis.

## Data Availability

The datasets supporting the conclusions of this article are included within the article (and its additional file(s)).
