# Supplementary material for: Assessment of a venous thromboembolism prophylaxis shared decision-making intervention (DASH-TOP) using the decisional conflict scale: a mixed-method study
Source: BMC Med Inform Decis Mak. 2023 Nov 6;23:250. doi: 10.1186/s12911-023-02349-3 (PMC10629184; doi:10.1186/s12911-023-02349-3)
Supplement: Supplementary file 1 — Additional file 1: Table S1. Open-ended questionnaire script for qualitative data collection. [file 12911_2023_2349_MOESM1_ESM.docx]

Table S1. Open-ended questionnaire script for qualitative data collection

| Individual interviews | | | |
| --- | --- | --- | --- |
| Question | Participants in **Group 1** responded to this question (Yes/No) | Participants in **Group 2** responded to this question (Yes/No) | Participants in **Group 3** responded to this question (Yes/No) |
| 1. What do you think this women would like to do to manage the risk of VTE during pregnancy, would you:  - Take low molecular weight heparin  - Without taking low molecular weight heparin  - Insecure about what to decide | Yes | Yes | Yes |
| 2. Why do you think this women have this preference? *(What factors have influenced the decision)* | Yes | Yes | Yes |
| 3. What type of information did you use to make your decision?  a) What information was most useful to you when taking the decision you made?  b) What information was least useful to you when taking the decision you made? | Yes | Yes | Yes |
| 4. Was the amount of information provided in the direct choice exercise appropriate? Why? | Yes | Yes | Yes |
| 5.Did you clearly understand the risks and benefits of heparin after completing the direct choice exercise? | Yes | Yes | Yes |
| 6. How do you think this women would balance the different aspects of the information presented(for example, the risks of having another blood clot compared to their personal experiences?  a) Could you explain your reasoning? | Yes | Yes | Yes |
| 7. Describe your experience with the rating scale and feeling thermometer | No | Yes | Yes |
| 8. Did these exercises help clarify your personal preferences for different health outcomes (for example, experiencing a blood clot)? | No | Yes | Yes |
| 9. Do you think these exercises reflected this women’s personal preferences? | No | Yes | Yes |
| 10. Was it helpful to have the results of the decision analysis model to make this decision? | No | No | Yes |
| 11. Do you think this women would only rely on the personalized decision analysis exercise to make a decision? | No | No | Yes |
